# Voluntary transition of the CEO: owner CEOs' sense of self before, during and after transition

**DOI:** 10.3389/fpsyg.2015.01633

**Published:** 2015-10-27

**Authors:** Randy T. Byrnes, Scott N. Taylor

**Affiliations:** ^1^Byrnes and Associates, LLCYork, PA, USA; ^2^Management Division, Babson CollegeBabson Park, MA, USA

**Keywords:** identity, role, role identity, transition, sense of self, CEO succession, role exit

## Abstract

This inductive study explores how former business owner chief executive officers (CEOs) experience sense of self during voluntary separation and transition from their company. Our inquiry engaged 16 CEOs who ran companies ranging in size from 15 to 500 employees as they detailed their stories of walking away from roles as owner CEOs. We developed a coding scheme to analyze themes manifested in the narratives. We also analyzed the former CEOs' narratives using a stage and valence model depicting both the continuum of the separation experience and the characterization of each stage as a positive or negative state of being. The diverse yet synchronous stories resulted in three implications for current owner CEOs, professionals who advise CEOs, and future research on CEOs' careers. First, the CEOs often failed to allocate sufficient time and effort to prepare for an identity shift following the sale of their company or transition into retirement. Second, the CEOs experienced a diminished sense of self and dissatisfaction with the exit event. Third, the majority of the CEOs demonstrated an ability to work through the adverse and unanticipated states of being into a positive sense of self.

## Introduction

“*On October 29, 1996 I signed papers to sell the company that I had owned since I was 23 years old. I was no longer the CEO of a regional staffing company of 48 dedicated professionals. My company was now ‘a division of …’ someone else's organization. I was instantly relegated to living off reputation rather than results; a spectator not a participant in the game of business; out of the club; off the team; a storyteller not a story-maker. No longer affiliated with a company (my company), I was adrift. Regardless of the financial outcome and the fact that I achieved at 45 years of age what most small business owners' work toward their entire lives, I found that I now had no response to the innocuous question—“So, what do you do”? The sale of my company was not a strategically planned culmination of a business career; nor was it forced upon me by age, unsatisfactory performance, or market circumstances. It was a conscious, opportunistic response to a desire for new, professional challenges and a belief that the decision would provide expanded career opportunities for my employees. The outcome was not what I had anticipated on many levels. In retrospect, the lack of preparation for the personal impact of separating from my company and coworkers was naïve and shortsighted”* (Randy, former owner CEO).

Given the aging baby boomer generation, over the next decade many chief executive officers (CEOs) and business owners will step away from the companies they now run and/or own. Of that group, small and mid-size businesses are a crucial element of the United States economy. The latest figures available report there are over 5.6 million businesses with less than 100 employees and of those, 5.1 million had fewer than 20 employees (The United States Census Bureau, 2012).

When you consider the number of small and mid-size businesses that will experience CEO turnover in the next decade, the implications for them, their organizations, and millions of workers they employ can be overwhelming. Legions of legal templates and financial formulas are available to business owners and their professional advisors to calculate estate taxes, construct succession plans, and facilitate a sale or departure by the CEO. What has received little attention is the *personal experience* of the owner CEO before, during, and after the process of voluntary separation and transition from the company (King, 2002; Wasserman, 2003; DeTienne, 2008).

Individuals who found a firm or own a majority stake in the enterprise frequently possess a heightened psychological and sociological connection to their company (Pierce et al., 2001; Wagner et al., 2003). How do owner CEOs prepare for an identity that is no longer founded on their work? When they transfer command and control of an organization, how do they locate or develop new role identities? What part, if any, does their sense of self have in these transitions?

The ambiguities and uncertainties present during this transition process can be great. The personal impact on the owner CEOs who sell their company could lead to introspective queries such as: Was I a hero in the business community for attaining the financial summit or a coward for taking the money and abandoning my co-workers? How will my family view me both during and following the separation process? When I no longer have my company, who am I? Their answers to these questions could impact how they leave and what they leave behind when they depart the organizations they lead.

Prior research has addressed CEO succession planning and transition (e.g., Biggs, 2004; Nadler et al., 2009) from the perspective of the organization, the challenges of retirement in general (e.g., Wang et al., 2011), and retirement's transitional implications (Beehr and Nielson, 1995). There is even emerging theoretical exploration that looks at the impact work-related identity transitions may have on an individual (e.g., Conroy and O'Leary-Kelly, 2014). We even know something of what CEOs experience when they retire (Kets de Vries, 1988; Sonnenfeld, 1991; Wasserman, 2003). For example Sonnenfeld (1991) created a typology to describe different styles CEOs assume upon retiring. He calls them: “Monarchs,” who do not leave voluntarily; “Generals,” who leave reluctantly; “Ambassadors,” who leave gracefully but retain close ties to their organization; and “Governors,” who leave willingly and pursue new opportunities outside the organization. Yet, none of these studies and typologies directly address the experience of transition from the perspective of the owner CEO and his or her sense of self during the transition. We see this as a gap in career transition research.

We hope to add to the growing understanding of the connections between role transition and identity (Ibarra and Barbulescu, 2010; Obodaru, 2012), but we also seek to broaden the discussion in the literature by looking at identity and role transition with a specific career role (owner CEOs) and career decision (voluntary transition). Thus, this study focuses on a work transition that is different from those traditionally studied. Ibarra (1999) and Pratt et al. (2006) have explored transitions at the *beginning* of careers, on the path to becoming bankers, consultants, or physicians. In contrast, this study focuses on a transition that involves a voluntary decision to *end* a career where one's identity is most often deeply connected to his or her role as an owner of the organization.

While our study and its implications may generalize to other senior executives or individuals who hold senior positions of authority, we chose to better understand the experience of the owner chief executive officers (hereafter referred to as simply CEOs) of mid-size to small businesses because of their importance to the economy and the lives of so many. The 16 former CEOs we interviewed represent multi-generation family businesses, small manufacturing firms, and nationally recognized service organizations. Their narratives provide a rich breadth of experiences regarding the sense of self in separation from one's company.

In sum, our purpose and this paper's contribution is to expand what we understand about career transition by selecting a sample of leaders who are rarely studied to shape theory. In doing so, we believe our work will suggest several imperatives for both research and practice to more effectively address the separation of leaders from their organizations.

## Literature review

The sense of self during transition is linked to the following areas in the literature: identity, role and role identity, transition and liminality, and anticipatory socialization.

These factors will in turn impact how a CEO seeks to establish a future outside of the role of CEO. A brief review of these literatures informs the assumptions we make at the onset of our study.

### Identity

Identity, as utilized in this work, is an aspect of the self-concept (Hattie, 1992) that addresses how people see themselves and are seen by others. Identity can be composed of two related concepts. A personal or subjectively referenced identity is how I see myself. A social or other-originated identity is how others see me and how I see myself in social categories (Tajfel and Turner, 1986). For the purpose of this study, “sense of self” incorporates both of these constructs. Self-categorization theory (Turner, 1975; Turner et al., 1987) proposes that individuals continually interchange personal and social identities. Tajfel and Turner (1986) describe this ongoing exchange as “relational and comparative” (p. 16). In other words, if one's social identity is salient then the individual will manifest the role behaviors held in esteem by members of a group (or category of people) to which they belong or aspire to belong.

The issue of salience, or one's readiness to act out a role or an identity, is important in our study because there are subjective and contextual influences that impact choices on identity and role selection (Ashforth, 2001). A CEO may select a role or identity that manifests behavior due more to the impact of an influential group rather than in service to the demands of the situation. For example, a CEO may take on the identity of “former CEO” by appointing an unprepared family member as the CEO's successor due to pressure from other family members, rather than any demonstrated competence by the successor. In our study, we are interested in how CEOs experience these identity decisions during their transition.

### Role and role identity

A role is a set of specific behaviors that are commonly recognized as typical and appropriate used by someone pursuing a particular goal (Biddle, 1979; Ashforth, 2001). To carry out a role one must possess the requisite qualities to perform the behaviors and have a purpose for enacting the role. The context in which the role is employed assists others with recognizing and identifying the role. Roles assist in understanding and developing perceptions and expectations of others and potentially provide opportunities for engaging in collective exchanges (Ashforth, 2001).

Roles may be created or assumed based on individuals' need to respond to the perceptions of others. If a CEO perceives a situation in which the appropriate role is to be a coach rather than an authoritarian leader, she would tend to select those behaviors that match the role of coach. In this way roles become context specific. As such, when shifts or challenges to habituated patterns arise, an individual may undergo a potentially dissonant experience. For CEOs separating from their company, they may be challenged to distinguish between the need to employ an established role or develop a new role. Whether the decision on role selection was made in reaction to a shift a CEO's sense of self or otherwise is an interest of our study.

Individuals with a role accrue an associated status and a set of expected behaviors and more of performance. They acquire a publicly defined way to be known. Roles develop from a sense of self and from one's foundation for identity (McAdams, 1993). The interaction of roles and identity is both a recursive and reflexive process. How individuals see themselves and their identity influences the roles to which they gravitate. Similarly, the roles people engage serve to affirm their sense of self (Ebaugh, 1988). A CEO spends considerable effort interchanging and appropriating roles such as decision-maker, mentor, adversary, advocate, and collaborator.

Role identity is that part of an individual persona (identity) that is composed of behaviors (roles) enacted to accomplish goals (Ashforth, 2001). Ashforth (2001) proposed, “A role identity [is] defined … as the persona associated with a role, including goals, values, beliefs, norms, interaction styles, and time horizons” (p. 264). He further clarified, “Role identities anchor and ground self-conceptions in social domains” (p. 27). However, a personal role identity and a social role identity may at times be dissociated.

The nuance of identity and identification is relevant to CEOs' differing perspectives on their psychological affiliation with their company. A CEO, when faced with a shift of role identity due to retirement or business sale, may confront affective, behavioral and cognitive challenges associated with the exit transition event. While it cannot be assumed CEOs will actively reflect on their identity and life trajectories (Langer, 1989), it is reasonable to assume they will engage in some meaning-making process. At times, they may be isolated and in other situations they may clearly desire the input of family or outside professionals to vindicate what their actions and feelings mean to their role identity.

Our study aims to understand how CEOs navigate the issue of changing roles and how this may impact their identity and role identity. When CEOs derive an identity from their company and then disengage from one role identity and reengage in some other role, are they able to recognize the shift? A transition from something implies a transition to something, even if that something is simply the absence of the old. How do CEOs close the gap between their old identity and a new identity?

In *Becoming an Ex*, Ebaugh (1988) studied role exit based on her personal, life-altering decision to leave a religious order. “The process of disengagement from a role that was central to one's self-identity and the reestablishment of an identity in a new role that takes into account one's ex-role constitutes the process I call role exit” (Ebaugh, p. 1). Residual identity is an important component of this role exit. Through the recognition of the individual's incorporation of the past in the reengagement transition, Ebaugh (1988) noted that regardless of the role abandoned, the individual retains a social identity from their pre-ex status. Whether a police officer or a convict, the individual's transitioning social identity never completely discards the previous classification. With this “hangover identity” (Wacquant, 1990) effect in mind, it is anticipated that former CEOs would manifest a residual identity in their reengagement process.

### Transition and liminality

Transition is the crossing of a boundary. The boundary may be psychological, physical, economic, social, or cultural. Regardless of the composition or the threshold of the boundary, transition is the experience of engaging and navigating a change from one perceived state to another. The concept of role transition has been described as role discontinuity (Benedict, 1938), status passage (Glaser and Strauss, 1971), transitions (Levinson, 1978), and passages (Sheehy, 1976). As individuals adapt to the changing needs of shifting social situations, they may alter roles to meet their contextual needs.

Several scholars have addressed the potential loss of one's sense of control and belonging in high intensity transitions (Latack, 1984; Ashforth, 2001; Conroy and O'Leary-Kelly, 2014). They propose that high intensity, high magnitude transitions have the potential to undermine the individual's capacity to manage ambiguity, uncertainty, or conflict inherent in the transition. If this state of high intensity transition describes the CEOs' experiences, it is reasonable to expect that our study's participants encountered difficulty navigating the gap in the exit process between established roles and the requirements of new roles and identities. This shift will challenge the executives' ability to process role and identity alternatives. The possibility therefore exists that the CEOs experience a state of liminality (Turner, 1969; Conroy and O'Leary-Kelly, 2014).

The presence of liminality, framed in this context as a psychological state, is understood to mean without a role identity or role-less-ness. “Liminality [is] the absence of a self-defining connection to a given social domain …” (Ashforth, 2001, p. 136). A number of theorists (Atchley, 1976; Beehr, 1986; Eby and Buch, 1995) view this condition of uncertainty in very positive terms. Ibarra (2003) highlighted the importance of engaging the unknown for transitioning CEOs or anyone else: “The *in-between period* is the crucible in which we bring our possible selves tentatively into the world. Unpleasant as it may be, we short-circuit it at our own peril” (p. 45).

In the liminal transition phase a CEO would be unsure which boundaries of their role identities to secure and which to cross. It is reasonable to anticipate hearing in the narratives about a period of time when the CEOs were without the social affirmation of their roles sets and had not yet assumed socially acceptable replacements. Conroy and O'Leary-Kelly (2014) theorized that this liminal period of transition can lead to a sense of work-related identity loss and an effort toward identity recovery. We are interested to see if the CEOs lived experience reflect either loss or recovery as theorized by Conroy and O'Leary-Kelly. In this regard, our study is one of the first to assess whether the transition experience of the CEO is one of cognitive and emotional processing of a loss orientation and/or a restoration orientation.

### Anticipatory socialization

Anticipatory socialization is the expectation of attaining a more satisfying role as motivation for role departure. The concept was originally formulated by Merton and Rossi (Merton, 1957a,b; Merton and Rossi, 1957). Someone engaged in this phenomenon will frequently acquire and internalize the values and orientations of a group to which the individual desires acceptance. In doing so, people project themselves into an enhanced state of being by abandoning a current role and replacing it with another role. Anticipatory socialization may encompass admission into a new collectivity or the assumption of a revised, independent identity associated with a new role.

Anticipatory socialization was noted in both Blau's (1973) and Ebaugh's (1988) theories of role exit. “People who voluntarily relinquish a role do so, as a rule, not merely because they are alienated from a role partner or role set but also because they have the hope or promise of greater satisfactions or benefits from some other role” (Blau, 1973, p. 211). Both theorists characterized anticipatory socialization as a bridge for the role-exiting individual to establish a revised social identity.

Anticipatory socialization is not without risks. If individuals aspire acceptance by a group that is unlikely to accept them, they may unknowingly create added anxiety or role strain in the exit process. An additional role strain can be experienced by the psychological impact of retirement and job loss. In such a case, a CEO may begin to more deeply consider the alternatives to leaving that were not taken (Obodaru, 2012). It has been acknowledged that how a person frames their departure has a direct impact on their emotional wellbeing (Lazarus and Folkman, 1984; Latack et al., 1995; McFadyen, 1995; Ashforth, 2001).

In sum, the above literature would suggest CEO departure, or any sort of similar career transition, will involve a shift in identity as both one's role and social context undergo dramatic changes. Such a shift will likely include a period of ambiguity and uncertainty (liminality) from which a new self should emerge (e.g., restoration orientation) reflecting identity stability. On the other hand, what we learn from the above literature only generally informs our understanding of CEO transition. None of the above works cited in the literature just reviewed examined CEOs directly. Given the potential overlap of their identity with their role (as owners and CEOs), we are interested in seeing how the theory and research just reviewed apply to our CEO sample.

Further, because of their impact on the lives of so many businesses, we feel it is imperative to understand how such transitions impact owner CEOs' identity and role as they experience separation and transition from their company. By doing so, we will be better prepared to help CEOs and others make these transitions more effectively, including those who advise CEOs (e.g., human resource professionals, consultants, executive coaches, etc.).

## Methods

### Sample

The sample of CEOs interviewed was limited to founders or owners who voluntarily sold or retired from their company within the last 20 years, and the company they led had an employee base >15 people. All those interviewed were former owner CEOs but not all of them were founders. This is important to clarify in that we are not conducting a study of entrepreneurs but of chief executives. To participate in our study the CEOs had to have held a significant or majority ownership position prior to their separation and were no longer in an operational, officer, director, or advisory capacity. This study was carried out in accordance with the Institutional Review Board (IRB) regulations of the first author's doctoral degree granting institution. As such, all CEOs interviewed for this study gave written informed consent in accordance with the proper IRB protocol.

The ages of the CEOs who participated in the study ranged from 32 to 78 years old. The average age at which they started as CEO was 39 and 58 when they exited. The sample served an average of 19 years as CEO. The size of companies at the start of the participants' roles as CEOs ranged from 1 person to 350 people. First-year revenues started below $50,000 to greater than $50,000,000, and CEO ownership percentage went from 11 to 100%. Company core management profiles ranged from individual CEOs to teams of parents and children. Finally, the exit pattern of 13 of the 16 participants was accomplished through the sale of their company; two retired and one CEO closed her company. In Appendix A (Supplementary Material), we provide a brief profile of each CEO interviewed.

### Procedure

The study methodology employs a phenomenological based narrative identity inquiry. Phenomenology is the study of the meaning of concrete human experiences (Slife and Williams, 1995) captured from the first-person point of view. In a phenomenological study, researchers interview participants and then “carefully analyze [the] accounts in order to understand not only the individual, private meaning of the experiences, but also what is general and illuminating in understanding the meaning of the human experience in a wider context of people and situations” (Slife and Williams, 1995, p. 200).

Since our primary aim was to understand the lived experiences of the CEOs from their own perspective (rather than that of their families or former employees, for example) and inductively learn more about owner CEO transition, a phenomenological narrative approach best met our purposes. CEOs interviewed were invited to retrospectively tell the story of their affective, cognitive, and behavioral experiences during their exit. Through these narratives emerge insights on how CEOs of small and mid-size businesses experience their sense of self through their role, identity, and role identity transitions during the separation process.

In terms of the actual interview itself, we used a narrative identity interview process (Clandinin and Connelly, 2000; Clandinin and Rosiek, 2007) wherein the former CEOs were asked to recount their lived experiences to enable them to construct their subjective stories of departure and transition from their companies. Appendix B (Supplementary Material) includes the interview questions we asked the CEOs. For the CEOs, the utilization of narrative identity acknowledges the individuality of their role identities within the context of the transition experience. Much of the CEOs' sense of self is in fact an effort at awareness of their *current* state of being. Because our focus is on how they conceptualized the past in the present, we did not have to be as concerned with whether the past they describe occurred within a few months or a few years. As noted by Weick (1995) “people learn their identities by projecting them into an environment and observing the consequences” (p. 23). We wanted to see how the CEOs would project their identities in the present.

### Analysis

To analyze the interview data, we used a thematic analysis (Boyatzis, 1998) approach, which is a process for encoding qualitative information. With thematic analysis, “codes” are created based on themes and patterns that emerge from the qualitative data. In addition to analyzing the CEOs' narrative interview data thematically and creating codes to represent patterns in the data, we also conducted a stage analysis, valence analysis, and sense of self analysis. The justification and our approach to each of these types of analysis are described below.

#### Coding of interviews

We first read each transcript and underlined statements that seemed relevant to informing us about the how the CEOs saw themselves and their roles in relation to their feelings, experiences, and decisions. These underlined statements were then collected from all of the interviews and analyzed for common themes across the interviews. In this case, a theme is “a pattern found in the information that at a minimum describes and organizes the possible observations and at a maximum interprets aspects of the phenomenon” (Boyatzis, 1998, p. 4).

From our initial coding efforts, we discovered the CEOs referenced their experiences by the stage of separation they were discussing. As will be discussed later, it became clear the CEOs referenced four distinct stages when describing their exit experience (i.e., Pre-separation Stage, Separation Stage, Transition Stage, and Present Stage). The initial coding also revealed strong positive and negative emotions related to the stage of separation the CEOs discussed (also discussed later). Finally, our coding found several general themes across the interview data. These themes were then consolidated into seven coding scheme groups.

Next, going back to the original interviews, the underlined interview statements were sorted according to the thematic groups to examine differences within the statements in each group. Because the data presented significant information related to identity, we next classified broad concepts about identity into a general Identity group. In doing so, it became clear the CEOs were discussing the concept of identity in several nuanced ways. As a result, we created three sub groupings for the Identity code (i.e., self-distinctiveness, self-enhancement, and self-knowledge). We present the coding scheme groups and their definitions in Table 1.

**Table 1:** **Coding scheme group definitions**.

**Group**	**Definition**	**Example**
Identity	Self-concept constructed by others (social identity) or formed by the individual and projected outward (personal identity)	“…my identity, as far as I was concerned was what I was doing in the community”
Self-distinctiveness	Sense of uniqueness or exceptional ability; voicing a perceived or demonstrable difference between one's view of themselves and of the sample in general or of their peer group specifically; highlighting outstanding or distinguishing features or experiences	“…I think how different I am from a lot of my peers, not just CEOs, but even just work day attorneys and engineers and whatever they may be …”
Self-enhancement	Self-promotion or confirmation of actions; affirmation of decisions; pride; satisfaction with oneself	“…reinforced my sense of self-worth, and that I was able to accomplish something”
Self-knowledge	Awareness of one's needs and capabilities; clarity of ability within a specific context	“ …guys like me, pretty much Type A personalities willing to take risks …”
Enhanced state of being	Positive perception of one's condition expression of happiness, peacefulness, satisfaction, looking forward	“Actually, I do have something I'm transitioning to that I think and hope will be interesting …”
Adverse state of being	Negative perception of one's condition; expression of disappointment, dissatisfaction, dissonance, regret, fear, failure	“ …I think then you really get to a point of without the connections and the people around and the need to be needed or the obligation to be somewhere, sometime then it gets really lonely and it's not necessarily a happy spot”
Presence of liminality	Sense of self-doubt, role-less-ness, uncertainty, apprehension, ambiguity, lacking a firm personal foundation	“But there is a sense of floating, a sense of, you know, now what”
Absence of liminality	Conviction of action, decisiveness, commitment	“I think that's true with a lot of business leaders, okay. We can have a long range plan and due to circumstances beyond our control—external circumstances usually—something comes along and every good businessman knows that you have to take advantage. Timing's everything. …And that's what happened here. I made the right decision at the right time”
Role	Behavior that is appropriate for pursuit of a goal and is context specific and assists in differentiating an individual in a social context	“…I spent some time thinking about that, what my options were, and really determined that I like to work. I like creating things, I like making something from nothing, I like creating value, I like, building stuff …”
Anticipated state of being	Future oriented; forward looking perspective; positive openness to an unknown condition, status or experience	“That was something I did and it's interesting and I enjoyed it, but it was not my whole life and there were other things that I thought were important …so I never really got so wrapped up in this that I never thought that life would end. But I guess in many ways I thought life would begin”

Subsequently, we calculated the frequency with which each of the seven thematic groups was mentioned in total and in one of the four separation stages. We calculated the frequency of mention of a given code based on total number of *participants* who made at least one statement relevant to that code, in order to more accurately reflect the breadth of a group in the study sample. Calculating code frequencies based on total number of *statements* relevant to that code could have reflected simply the code's saliency to one or two individuals. The frequency data is presented in Table 2.

**Table 2:** **Number of people mentioning each coding element by stage**.

**Group**	**Total number of people**	**Stage^a^**			
		**A**	**B**	**C**	**D**
Identity	12	8	4	5	2
Self-distinctiveness	8	3		7	
Self-enhancement	13	4	2	8	3
Self-knowledge	10	4	1	8	4
Enhanced state of being	12	2	8	6	3
Adverse state of being	11	1	7	7	1
Presence of liminality	8	1	2	4	2
Absence of liminality	12	6	4	7	1
Role	7	1		5	1
Anticipated state of being	3	2		2	
Total frequency of group coded statements by stage		32	28	59	17

a *A, B, C, D represent the four stages: A, Pre-Separation Stage; B, Separation Stage; C, Transition Stage; D, Present Stage*.

Some may question why we decided to report frequencies, rather than categorizing participants themselves into thematic groups and describing respondent profiles beyond what we present in Appendix A (Supplementary Material). The reason for our decision is based on allowing the data and themes derived to dictate our analyses. The data itself was saturated with the seven common themes more so than presenting a deeper biographical sketch of the person. Since our primary research question was how CEOs described their sense of self during and after transition, we found the data to support our effort to focus on the seven thematic groups.

Finally, in order to characterize the state of being of each participant during the four stages of separation, we assigned a positive or negative overall valence to each stage for each participant (to be discussed later).

A second coder was engaged to ensure the reliability of the coding process. The second coder was trained in the themes and then asked to code the 16 transcribed interviews. With regard to determining in which Stage a given statement was referenced, 77% were coded for the same Stage by the coders. Of the valence ratings possibilities, the second coder and the first author had 73% initial agreement. The second coder did not select a valence for one stage of one CEO. The greatest discrepancy in the second coder reliability process was the application of Presence and Absence of Liminality. This will be addressed later. The agreement rate among the two coders reached 100% for all of the statements after discussion.

#### Sense of self, stage, and valence analyses

Given the initial results from our coding, three additional types of analyses were conducted: a sense of self analysis, a stage analysis, and a valence analysis. The impetus for these were completely derived from the coding process and therefore were data driven (Boyatzis, 1998).

##### Sense of self analysis

In the sense of self analysis we assessed how many times participants mentioned each coding group within each of the four stages (i.e., pre-separation, separation, transition, and present) of their exit experience. Using this framework we sought to understand which coding groups were mentioned the most by participants (see Table 2).

##### Stage analysis

Prior literature on the identity transition process has focused primarily on a separation phase (Ebaugh, 1988; Ashforth, 2001) and a transition phase (Ibarra, 1999; Ashforth, 2001; Ibarra and Barbulescu, 2010; Conroy and O'Leary-Kelly, 2014). We assumed our CEOs would focus on these two phases (separation and transition); thus, our interview questions reflect this assumption (see Appendix B in Supplementary Material). On the other hand, in our analysis of the interview data it became clear that the CEOs were actually describing four stages of their experience. Specifically, in addition to separation and transition, the CEOs consistently discussed their experience leading up to separation and their life after separation. Hence, we began coding the data accordingly. From the transcribed interviews, events described were separated into four stages or time categories: Pre-separation Stage (events occurring before the separation or company exit event), Separation Stage (events that occurred during the separation or exit event), Transition Stage (events following the separation or exit event), and Present Stage (events occurring in the present time of the interview). Our last stage, the Present Stage, captures not only the condition of the individual at the time of our interviews but also reflects what we consider the resulting identity after transition (referred to as the “reincorporation stage” by some, e.g., Conroy and O'Leary-Kelly, 2014).

##### Valence analysis

Markus and Nurius (1986) utilized the term valence in reference to transition. We adapted their strong-to-weak valence continuum into the concept of a positive and negative valence to characterize the state of being of each participant during the four stages of separation. The use of positive and negative designations was not a moral or personal judgment on the person but characterizes the individual's description of their sense of self at that time. The positive/negative designations were intended to depict the tone of the person's comments during each period.

## Results

### Sense of self analysis

The Transition Stage included the most coded statements (110) while the Pre-Separation and Separation Stages had 51 and 40 coded statements respectively. The Present Stage had 25 individuals' coded statements.

Based on the criterion of the number of individuals for whom at least one statement was coded, seven coded groups were kept for analysis and discussion: 1, Identity; 2, Enhanced State of Being; 3, Adverse State of Being; 4, Presence of Liminality; 5, Absence of Liminality; 6, Role; and 7, Anticipated State of Being. As noted earlier, the identity grouping had three sub groups: self-distinctiveness, self-enhancement, and self-knowledge (see Table 2).

#### Identity

Statements coded as Identity included social identity and personal identity statements. Social identity (or self-concept constructed by others) was less frequently mentioned (six participants) than was personal identity (11 participants). We proposed earlier that leaders of organizations were aware of external judgments from others, such as customers and employees, but ultimately depended on their individual beliefs in decision making. The statements by the study participants support this observation. While acknowledging others perspectives—“… most people from the time I started thought I was crazy” (Mike)—they were not likely to spend much time seeking affirmations: “I care more what I think about myself than what other people think about me” (Nick).

Statements about personal identity included former CEOs who professed a connection between their personal identity and their company (three participants) and those who noted a separation between themselves and their company (six participants). The comments about connection or lack thereof between personal identity and one's company were expressed more frequently in the opening remarks of the Pre-separation Stage than in other stages. Witness the contrast in the following perspective of an individual's self-concept whose identity was independent of his company with a participant whose personal identity and the company were one:

“I had done enough things outside the company that the company was not who I thought I was.” (Abe)

Versus:

“…if you're going into effect a retirement like I did, you're going from being a big deal to just an average, another person on the street. You don't feel needed. You don't feel connected.” (Carl)

Stating an attachment to one's company was characterized both positively and negatively in the valence analysis. Pat, who claimed, “I was the business. The product was me” was also one of the three participants to describe all four stages positively.

#### Self-distinctiveness

Eight of the participants in the study expressed their uniqueness both in a role identity comparison to their peers and with respect to a personal identity. The category was heavily weighted (seven of the eight individuals) to the Transition Stage.

“What we did was not typical, but what does the entrepreneur do that's typical?” (Nick)

“I think how different I am from a lot of my peers.” (Greg)

“I'm pretty sure that I'm not very typical.” (Abe)

“I had this platform, this star quality that I was able to use for good.” (Hank)

The context in which these remarks were made varied and therefore no further inference is drawn other than to note their existence by half of the interviewees.

#### Self-enhancement

Self-enhancement statements were coded for 13 of the 16 participants. Interestingly, a preponderance of these comments (eight individuals) was made in the Transition Stage following the separation event. Participants chose this stage of their narratives to reflect on positive outcomes from their decisions or to recognize their value to the company from which they departed.

“…without sounding too egotistical, I really was a strong point in the business.” (Kate)

“I find it difficult to criticize many of the decisions I made …” (Otto)

Whether justifying, rationalizing or stating the truth as they remember it, self-enhancing statements were most frequent in the stage that had the highest overall negative rating (Transition stage). These statements seem to presage a working through of adversity described by many and that working through eventually led to the more positively rated final, Present Stage. One of the participants, after considering the impact of the loss of title and potential negative repercussions as a community leader, resolved his quandary with the self-enhancing observation “…I don't need to have all the credentials to get in the door. I'm here, let's play.” (Donald).

#### Self-knowledge

Self-knowledge manifested itself both as a reflective awareness and an acknowledgment of what individuals were now cognizant of going forward. Lessons learned about oneself as a result of the separation experience were expressed by just less than half of the16 individuals.

“I'm tougher than I used to be. I can do anything.” (Lucy)

“I'm not going to sacrifice relationships, family …just to make money.” (Otto)

“If I didn't have that courage (to fail) I would never have been anywhere.” (Mike)

The individuals who leveraged their separation and exit experiences to restructure personal priorities, and in some cases undertake completely unrelated new career ventures, made statements such as, “…there's a lot of different places you could (go), I guess. But finding something that really matters in this case to me is making the social or environmental contributions …” (Carl). In Ibarra's (2003) terms they were exploring their “possible selves.”

#### State of being and sale transaction

Eight participants in their descriptions of the sale transaction mentioned enhanced and/or adverse states of being. Six individuals referenced both negative (adverse state of being) and positive (enhanced state of being) comments with regard to preparation for a sales transaction and subsequent exit event, while two individuals mentioned negative comments only and two individuals mentioned positive comments only.

In the adverse state, eight participants expressed difficulty with both the transaction process and their sense of self.

“…the idea of selling, it was just a grief-wrenching thing, I just felt, like somebody had died …” (Lucy)

“…once in a lifetime scenario to meld my belief system into a business—I let it go …very difficult to come to grips with—letting go …” (Hank)

“I had several emotions—biggest one was a sense of failure.” (Otto)

The eight interviewees who expressed a positive perspective of their affective state with the closing of a transaction noted both relief and a sense of accomplishment.

“…what I would call a bit of euphoria after the deal was done and closed and the funds were wired and sort of that actual transition happened. And that burden, that responsibility had been lifted and I now was financially set.” (Greg)

“I was excited when it happened.”(Kate)

“…the high point, my daughters said to me, ‘Dad, this was the right thing.’” (Jim)

In general, the adverse state of being frequently preceded a sale process and an enhanced state of being often followed a sale completion. On the other hand, the CEOs who took over new roles as a company employee did not experience an enhanced state of being following the sale of their company, mentioning negative feelings about their revised relationships with former employees. Given the importance of the separation event, it was quite interesting that six of the participants did not reference their emotional state in any manner regarding a sale transaction or retirement event.

#### Liminality

Fourteen of the 16 participants made statements in their narratives that qualified as either the presence or absence of liminality. The former CEOs and their non-liminal mindset were typified in a comment by Jim, “But you have to understand, I'm a businessman and got to close the deal.” When faced with the competing priorities of self, family, shareholders, employees, vendors, bankers, and others, the CEOs in this study weighed their options and made their decisions. There was little in-between ground. On the other hand, those who manifested the presence of liminality in the interviews did so primarily during the transition stage.

#### Role and anticipated state of being

Role and anticipated state of being were combined in this analysis because there were very few anticipated state of being statements (discussed later), and most of them referenced new roles. In the Pre-separation Stage there were comments about looking forward to a state of financial and personal freedom, “I thought it would be really fun to take advantage of having liquid wealth …” (Abe) and “…I'll have this many hours each week that I can divide between family, etc.” (Greg). In the Transition Stage many of the anticipatory comments alluded to the taking or making of roles.

“I thought I would enjoy venture capital, angel investing …” (Carl)

“I looked forward to my daughters taking over the business. I looked forward to coaching them.” (Jim)

We found it unusual that individuals who were tasked with handing over the future of their company would acknowledge so little awareness of planning for their own future separation from their firm.

### Stage analysis

The Pre-Separation Stage served as the relationship-building foundation of the interview process. Although our research question focused on a sense of self during separation, it was necessary to establish a safe environment for the participants in which the former CEOs could feel comfortable describing their thoughts, feelings, and actions during a high magnitude and high intensity time period for them. This was accomplished by initially asking each of them to tell us how they came to the CEO role.

By recalling the familiar story of their foundation in business they were able to segue into more intangible self-perception descriptions during the Pre-Separation Stage. The participants referenced their immersion into leadership roles and the associated trials encountered in building a company. The connection or separation of one's personal identity and the company was noted. They recalled affective states with “I felt …” introductions and forged into the first nuanced descriptions of both positive and negative experiences and corresponding unanticipated states of being that opened the way to the Separation Stage.

Our attention in the Separation Stage was drawn to the adverse and enhanced states of being expressed. Following the sales transaction, five of the participants accepted roles with an acquiring company. Their sense of self, personal identity, and social identity were universally negative. This unanticipated state of being was intense, and this experience accounted for five of the eight individuals for whom the Separation Stage was negative. Forced to address not only their own sense of role identity but also their altered and sometimes damaged relationships with former employees raised the stakes in their efforts at regaining a self-concept that met their needs. This phase provided options for navigating the Transition Stage. Individuals could find a way through the altered role identities and contingent relationships, or they could succumb to the psychological burden of ongoing retroflection.

What struck us as most interesting in the Transition Stage were the three identity sub groups of self-distinctiveness, self-enhancement, and self-knowledge that seemed paradoxically prominent in a stage that was rated as having a negative valence for 10 of the 16 participants. This period was the most reflective for the participants as they often engaged in overt sensemaking activities as their narratives unfolded.

Lucy typified the recursive impact of simultaneous storytelling and sensemaking in an exchange near the end of our interview. She stated that she actually realized during our conversation that she had developed a sense of confidence in her ability to learn new skills that she had never before acknowledged. The awareness and sensemaking through narration episode was a striking example of a benefit of storytelling methodology.

The Transition Stage covered timeframes from 3 months to multiple years. As the former CEOs simultaneously constructed and reacted to their stories, they demonstrated the ability to rehash events and outcomes. This rehashing activity we believe contributed to the intensified critique of themselves and their experiences during this time. Paradoxically, while giving voice to this criticism, they concurrently made the greatest number of self-enhancing remarks. The duality, we suspect, served to support a working-through process of this stage. The ongoing crossing of boundaries both figuratively and literally in the Transition Stage established the foundation for movement to the Present Stage.

The participants' sense of self connection in the Present Stage was clearly enunciated with statements witnessing resolution of personal and social quandaries and positive reflections on efforts made and outcomes achieved. Family issues, if contentious at one time, were treated as resolved. Disappointments were characterized as dealt with, and affirmations of decisions made in the best interests of all were staked as truth. The confidence to move forward into new roles and undetermined futures was alluded to by multiple interviewees.

Recognizing the unresolved, negatively depicted Present Stage states of three of the participants magnified the working-through process and resolutions achieved by the other 13 members in the study.

### Valence analysis

Table 3 reflects the results of analyzing the valence of each participant at each stage of separation. For example, Table 3 shows Nick moving from a positive (+) Pre-separation Stage to a negative (−) Separation Stage. We inferred this shift from his Separation Stage comments such as “…when I did exit, you still lose a piece of yourself” and “…try not to be overly emotional but this (exit) gnawed at me.” We interpreted these statements as a negative self-review of the individual's circumstances. The primary result from this analysis shows that 10 of the 16 participants transitioned from a negative to a positive valence characterization of their state of being or sense of self at some point during their narratives (see Table 3).

**Table 3:** **Participant valence on sense of self in each stage**.

**Stage**				
**Totals**	**A**	**B**	**C**	**D**
	**16**+ **0**−	**8**+ **8**−	**6**+ **10**−	**13**+ **3**−
Abe	+	+	+	+
Boris	+	+	−	+
Carl	+	+	−	−
Donald	+	+	−	+
Ed	+	−	+	+
Frank	+	−	−	+
Greg	+	+	−	+
Hank	+	−	−	−
Ike	+	+	+	−
Jim	+	−	−	+
Kate	+	+	+	+
Lucy	+	−	−	+
Mike	+	−	+	+
Nick	+	−	−	+
Otto	+	−	−	+
Pat	+	+	+	+

*A, B, C, D represent the four stages of separation: A, Pre-separation Stage; B, Separation Stage; C, Transition Stage; D, Present Stage*.

As seen in Table 3, three of the 16 participants described a positive sense of self throughout all four of their stages; six described a positive perspective in three of their four stages; six described two positive and two negative stages; and one person described three out of four stages as negative. Interestingly, no one described four negative stages. This generally positive characterization (43 of 64 total stage segments or 67%) may be due to the speakers' propensity in storytelling to reconstruct their story over time and build narratives that were more personally pleasing to both narrator and audience. However, there was no way to know since this study was not designed to test the veracity of the individuals' recollections but accept them as the participants' truth.

## Discussion

The methodology for a phenomenological narrative study permitted us to explore the CEOs experience through story as the foundational communication tool. Given the breadth of results, we found three key findings: (1) 13 of the 16 participants did not describe any awareness of an anticipated state of being or looking forward process for the period following exit from their company, (2) 11 of the 16 participants encountered an adverse state of being during or following their separation that undermined much, if not all, of their satisfaction at completing the sale event of their company, and (3) 10 of the 16 participants transitioned from a negative to a positive valence characterization of their state of being or sense of self at some point during their narratives (see Table 3). This could be described as a working through adversity or challenge.

### Unanticipated state of being

At the outset of this study we viewed the interaction of sense of self and the CEOs' separation as a static event. We came to understand it as a dynamic process. The development of the four stage valence model allowed us to expand what we envisioned as a singular experience into a more system-like iterative interchange of emotions, thoughts, and actions.

Anticipated State of Being was the least frequently mentioned coded group in the study. It struck us as remarkable that only three of 16 former CEOs enunciated any awareness of an anticipated state of being or projecting themselves into the period following exit from their company. The corollary that the remaining 13 former CEOs failed to acknowledge what their life might be following separation seemed incongruous with strategic thinking skills often associated with the CEO role. This might be explained by the CEOs' assumed propensity for successfully adapting to changing business conditions and unforeseen challenges over time. Possibly their predilection for data gathering and making decisions gave them a sense of confidence in their capacity to adjust to new roles following an exit event. Another possible explanation may be they generally underestimated the complexity and magnitude of the role set changes and as a result found themselves unprepared for the repercussions of the separation.

On the other hand, given their level in the organization as CEOs, could it be they had less opportunity to have narratives with others (i.e., “lonely at the top”) to navigate future alternative identities? More specifically, Ibarra and Barbulescu (2010) state that identity work consists of “people's engagement in forming, repairing, maintaining, strengthening, or revising their identities” (p. 137). They introduce narrative identity work as the “social efforts to craft self-narratives that meet a person's identity aims” (p. 137) and propose that “narrative identity work will be more prevalent the more the work role transition interaction episodes are high stakes, are visible, and/or concern role set members” (p. 140). In the case of these CEOs, is it possible they are limited by their role and profile to engage in the very narratives that would help them begin constructing alternative identities to replace their current one as owner CEOs?

Alternatively, recent theorizing by Conroy and O'Leary-Kelly (2014) focuses on the separation stage and the emotions and experiences of the individual during this stage. They propose that the dominate type of emotion one experiences in transition will influence the extent to which the person will reach out for social information to aid them in identity transition. What is not adequately accounted for in their work is an examination of what we called the Pre-Separation stage and the emotions one feels in that stage in comparison to the Separation and Transition stages. Our results found that *all* 16 CEOs had a positive valence in the Pre-Separation stage. Perhaps the lack of discussing a future, anticipated state of being relates to the stark contrast in valence experienced in the second and third stages compared to the first. We believe this is a critical area for further study. First, future work should not simply focus on separation and transition stages but also consider pre-separation. Second, further work should see if when there is a strong valence difference between pre-separation and the other two stages. If so, does this limit the degree to which leaders even consider seeking social information altogether; thus making the separation and transition stages more negative?

Our results seem to suggest that alternative identity construction will be impacted by the magnitude of the transition, not just from the separation stage but possibly also from the pre-separation stage. In particular, this may impact not only the ability but even the awareness of the need to engage with others about alternative identities. A CEO's developmental network is a difficult challenge as others are more inclined to tell him or her what they think the CEO wants to know rather than we she or he needs to know. This combined with the CEO feeling positive in the Pre-Separation stage may lead to CEOs not engaging in narratives about their anticipated state of being during transition and separation.

### Adverse state of being related to separation

The missing sense of self foresight may have contributed to the second finding that 10 of the participants described an adverse state of being following their separation. This undermined much, if not all, of their satisfaction at completing the separation event. Of the seven adverse state of being participants in the Transition Stage, five were impacted by negative contextual circumstances of changing roles within their companies following a sale. The role set (Merton, 1957a) of behaviors each had to acquire as an employee of the acquiring company proved difficult. This challenge was a combination of adapting new duties and reporting responsibilities along with experiencing altered relationships with former employees. Three additional participants who did not take positions with the acquiring company also expressed adverse sense of self (with regard to former employees) following their separation.

Bridges (2003) stated that the starting point of any transition actually begins with acknowledging the end point of the old situation. In the case of the five former CEOs who became employees, the alteration of authority and responsibility levels in their new positions may have appeared so nuanced to both the CEOs and employees before the transaction that neither group fully appreciated the magnitude of the change until it was upon them. This resulted in the former employees' inability to accept an altered role set for the CEO-now-coworker. The reconfigured role identity for the CEOs also revised personal and social identities. The description of the new state of being by the CEOs was generally negative and unanticipated.

As noted by Ebaugh (1988), regardless of the role abandoned, the individual will frequently retain a social identity from his or her pre-ex status. The retention of a CEO role identity combined with the intensity and magnitude of the professional status change in the leaders' role set made for potential loss of their sense of control and belonging (Latack, 1984; Ashforth, 2001). In each case, it was not until the CEOs aborted or renegotiated or completed their employment agreement and separated from the company that they described their situation and sense of self in a positive frame.

Liminality was identified by Turner (1969) as a sense of role-less-ness or without an identity. This was not the case with these CEOs as much as a personal disconnect with the parameters of the new role established by the employer. The dissonance between their previous role identity and the revised role identity following separation impacted their sense of self with surprising intensity. The former CEOs in this study often did not manifest an awareness of the reasons behind changes in roles as much as expressing an action orientation to doing what they saw as required at the time to adapt to a changing environment.

The relationship between the CEOs' altered roles and their identities was referenced by McAdams' (1993) contention that as the individual acquires roles there is an associated acquisition of status. When the status or public way to be known is altered, one's sense of self is also altered. This was the case for the CEOs who transitioned from owner to employee in their separation process. The CEOs' attempt at *role making* (the creation of a new role) described by Ebaugh (1988) was universally unsuccessful. Again, we suggest this may be due to a limited opportunity to construct alternative identities which could then assume possible identities to explore. Ibarra and Barbulescu (2010) have noted that people struggle emotionally when they are unable to make a link between their old self and their new self toward a more permanent sense of self. Conroy and O'Leary-Kelly (2014) call this more permanent self the point of “identity stability.”

The connection between old and new selves manifested itself not only in the agentic roles of the five executives who became employees but also in their new relationship with their former employees. This communal component of shifting identities and roles was addressed in Ashforth's (2001) work on role identities interlocking component with employees. The former executives' role sets to which they had become accustomed and were socially validated by the employees were now in dissonance. When the owner CEOs lost their social capacity to affirm their CEO role, they lost their identity (i.e., identity instability). As a result, the CEOs and their employees found the leaders in unfamiliar role sets lacking authority to address wrongs and resolve questions. The repercussions of the ensuing role set dissonance were diminished enthusiasm for the state of being following a sale transaction.

### Negative to positive valence characterization

All of the 16 former CEOs began their narratives with a consensus positive characterization (Pre-Separation Stage). This shifted to a 50/50 positive/negative experience as they described their preparations for an exit event, executed a sale transaction, or adjusted to unforeseen immediate consequences of their separation decisions (Separation Stage). The sense of self evolution was followed by a further negative shift in the Transition Stage as roles changed, role sets required alterations, identities were juxtaposed, and boundaries were crossed. The 10 participants who transitioned from a negative to a positive state in the course of their separation and transition process provided a model of “working through.”

In an effort to better understand what dynamics might have been at work for the participants, we returned to the data and looked at the comments made by each of the eight participants who shifted from a negative rating in the Transition Stage to a positive rating in the Present Stage. We reviewed their statements in both stages. Seven of the eight former CEOs had actively assumed new roles *of their choosing*. In some cases they experimented with alternatives such as commercial real estate management or becoming a mentor of nascent entrepreneurs. In others it was a fluid transition into retirement and an easing into a new recreational identity.

Ibarra and Barbulescu (2010) describe the concept of macro role transitions regarding a process of adaptation and change. Their contention (in contrast to Bridges, 2003) that significant transitions “may begin long before an actual role change and often extends beyond it” (p. 137) could help to explain the working-through process. Possibly the CEOs possessed an understated awareness that their roles would change following an exit event and there was an assumed confidence that the process would be successfully managed. With such a belief in oneself, there might have been a tendency to presume positive outcomes. In the specific scenario of separation from one's company due to a sale transaction or retirement event, it is reasonable to postulate that each of the CEOs knew intuitively a termination event was probable. Potentially the acknowledged background of their inevitable separation supported or required a pervasively positive perspective in the storytelling of their exit. Regardless of the difficulty in pinpointing definitive correlations between the CEOs' post-exit decisions and the positive characterization of their separation, a working-through process materialized.

What we did not adequately capture in our interviews is how this working-though process occurred in the experience of the CEOs. Conroy and O'Leary-Kelly (2014) comment on part of what we heard in the CEOs' narratives when the two researchers address the movement from one work identity to another as movement from a loss orientation to a restoration orientation. This period is fraught with emotional instability, which our results captured in the valence analysis. On the other hand, in the researchers' theory, restoration orientation is characterized by individuals who are “forward looking, striving to determine who they will become” (p. 77). Given the positive valence for all the CEOs in the Present Stage, we assume they experienced a point of restoration at some point. But our data did not capture when or how this restoration occurred, either because the design of our Interview Guide (in particular questions 7–9) was not able to capture that information, or the CEOs themselves did not experience restoration in the way proposed by Conroy and O'Leary-Kelly.

In the Present Stage, four of the participants had moved into a retirement role, one person was in a state of liminality regarding the next role, and the remaining 11 transitioned into alternative leadership roles in new enterprises or community-based organizations. For those executives who actively pursued new roles and role identities, there was an added manifestation of leveraging some of their leadership attributes in the form of residual identity. Similar to the women in the Breese and O'Toole (1995) study who specifically noted the integration of their strengths and needs into new roles as students, the 11 former CEOs constructed new roles from their known aptitudes. Stated by Boris, “I think it (new professional role) will be interesting and intellectually stimulating. People who know me tell me they think I'll be good at it …It's me.” These individuals were taking or making roles that leveraged their expertise.

Johnson's (2003) work with 12 former school principals' experiences of role exit was a variation of the residual identity concept. This was expressed by Johnson as role legacy. The majority of her participants vocalized a need to confirm that they had been good principals and they had been effective leaders in their schools prior to departure. Ten of the former CEOs manifested their version of role legacy with their social affirmation comments: “It was more comfortable once I found that employees didn't think as bad of me as maybe I thought they did. So it became more comfortable” (Frank) and “Really I'm not being an egotist, but I have to be proud of myself for what I did” (Mike).

## Research limitations

Due to its inductive, exploratory nature, this study had inherent limitations. First, the tightly defined demographic of small and mid-size company owner CEOs may reduce the applicability of the findings to a more diverse sample. Likewise, our sample presented a primarily male group with a wide range of ages. This too raises questions about the generalizability of our results.

Second, the selection process for the participants was not random as the former CEOs were screened for inclusion based on company size and additional criteria noted earlier. Nonetheless, with their demonstrated capacity to lead and influence, the CEOs offered a rich foundation for better understanding how transition of significant career changes might be managed. Their lessons learned have application to both the role exit and career transition research.

Third, while the use of narrative for inquiry is well established (McAdams, 1993; Josselson and Lieblich, 1999; Denzin and Lincoln, 2003; Rubin and Rubin, 2005), the reliance on retrospection for understanding has noted limitations. Likewise, our data was self-report, could be influenced by the amount of time since CEO exit, and was single source of data. These issues can impact the validity of our findings.

## Implications for future theorizing

Our study reveals three observations related to CEO transition and employee transition in general that should be considered in future theorizing. First, the CEOs often failed to allocate sufficient time and effort to prepare for a personal state of being shift following the sale of their company or transition into retirement. Second, the CEOs experienced a diminished sense of self and dissatisfaction with the exit event. Third, the CEOs demonstrated an ability to work through the adverse and unanticipated states of being into a positive sense of self.

The sense of self or self-concept construct utilized by identity theorists is centered on the argument that one's identity is grounded on the perception of others (Mead, 1934; Stryker, 1980; Schlenker, 1986; Landfield, 1988; Burke, 1991). In this study, in general, that was not the case. At the same time few of the theories just cited were built on observations of individuals like the sample we studied. In fact the CEO population has been woefully understudied, mostly due to their inaccessibility.

Exogenous frames of reference regarding self-worth or satisfaction with one's performance were noted by the five CEOs who remained with the acquiring company following the sale of their firms. They commented on the importance of former employees' perceptions of them in their revised roles. This was not a consensus experience. While an awareness of external impacts was not common to all of the participants, there were acknowledgments by the former CEOs of others' attempts to categorize or associate them with specific social groups, but there was little impact noted on their sense of self.

Ashforth (2001) suggested that self-categorization occurs when individuals are aware of the contextual and personal influences present. In this study, the participants manifested a capacity to interchange personal and social identities by developing new roles (Ebaugh, 1988), especially in the Transition Stage following separation. As they recognized the impact of the separation event on their roles and relationships, there were corresponding shifts to new role development or role assumption.

McAdams' (1993) position that roles develop from a sense of self and form the foundation for identity was connected to the process of new role formation in this study. With regard to new roles, it could be argued that the CEOs who initially remained with the acquiring company assumed or *took* an established role. It would be more accurate to describe their revised roles as integrations or *made* roles in the new contexts. Ebaugh's (1988) differentiation of role-taking and role-making actions was an accurate depiction for the Transition Stage. An interesting aspect of the role-making was how few of the participants knew what the new role would be. Almost to a person they planned to “make that up as I go” (Boris). The ironic aspect to this “wing it” mentality was its paradoxical perspective with their exit advice to future CEOs. Their exit advice comments were filled with admonitions about establishing a personal plan prior to any separation event. Do as I say (now that I've learned), do not do as I did.

Anticipatory socialization was referenced earlier in this study in Blau's (1973) and Ebaugh's (1988) theories of role exit. They hypothesized that individuals often shifted roles because they aspired to some anticipated benefit of inclusion in a new group or crossed role boundaries due to the desire to exit a current position. This was generally not the case with this sample. The majority of participants undertook a sale transaction or retirement process not primarily to join a new group or exit their current role but to maximize a financial window of opportunity. The new roles or groups available to them as a consequence were secondary in their narratives.

Although anticipatory socialization was not the driver for crossing role boundaries, the former CEOs did undertake steps to locate themselves in revised social contexts following separation from their organization. Some aligned themselves with like-minded individuals who were retired; others immediately started new ventures to remain within a valued cohort of entrepreneurs. Ashforth and Mael (1989) noted individuals' propensity to classify themselves into cohorts and collectivities of similar interests. The effort to establish both an individual persona and affiliation with a desired collectivity served to support the bridge-crossing experience of changing role identities. Through affiliation with a group, these individuals joined or created a category with which to distinguish themselves in the social environment. This grounding exercise established the basis for answering the fundamental question, “Who am I”?

Similar to Van Steenberg's (1988) work on exiters from large corporations, this study cohort did not align exactly with Ebaugh's (1988) work on role exit and her four stages: doubts, seeking and weighing role alternatives, turning points in the role exit process, and post-exit adjustment or the establishment of new roles and identities in the post-exit period.

Although the former CEOs experienced a voluntary exit, their narratives also did not include commentary that we could segment into the definitive stages identified by Ebaugh and Ashforth (2001), which suggested that the four stages be treated as tendencies rather than fixed stages.

There are reasonable hypotheses why the participants' narratives did not exactly align with the Ebaugh stages. Possibly, the difference between the *total lifestyle* role change that Ebaugh (1988) studied and the more circumscribed *professional career* role change that we studied might account for the differences in these results. Also, the individuals in Ebaugh's work were often considering significant alternative lifestyles such as former religious or former convicts exploring heretofore unimaginable new roles and identities. The CEOs may have seen their revised lifestyles as a more moderate extension of their current self-concept and therefore did not allocate significant time to seeking and weighing new role alternatives. Lastly, although self-doubts were acknowledged by a few of the CEOs in this study, they frequently pertained to the outcome of the sale transaction's impact on employees rather than trepidation regarding their personal lifestyle roles.

## Implications for future research

We suggested that the inability of the CEOs to navigate the shift in their identity may have resulted from a lack social effort to engage in narratives that would help them construct a new identity post transition (Ibarra and Barbulescu, 2010). We see this as a key area of future scholarly exploration for not just CEOs but those who are seen as senior leaders in their organizations. Those “at the top” may be limited in people they can confide in to have identity construction conversations. Or, alternatively, are those at the top less inclined to want to engage in such conversations? Given the magnitude of the transition (i.e., leaving the “helm”), it is likely that the lack of such narratives for either reason will impair their transition, especially in identity restoration (Conroy and O'Leary-Kelly, 2014). Future research should explore the potential barrier that may impede such identity defining discussions from occurring. We suggest using Conroy and O'Leary-Kelly's (2014) model of work-related loss and recovery as a guide to formulating interview questions that will better access to what degree and with what method senior leaders approach identity and role restoration.

As noted earlier, prior work documented that most founders do not want to give up control or leave their “baby/mistress” to others to care (Kets de Vries, 1988); even those who have sold the business tend to stay with their firm in other roles (Wasserman, 2003). These 16 owner CEOs appear to be different. An interesting question to ask is why the difference? Our findings offer some possible answers, but more research needs to be conducted. For example, we question whether there has been a shift over the last decade (or longer) to CEOs having a shorter term focus and an increased drive to seize personal economic opportunity when present. Given the general shorter tenure of CEOs in today's hyper competitive environment, perhaps “holding on” is less of an issue than in the past. Similarly, could this be why these owner CEOs entered such a major transition, both for themselves and their firms, without much planning about its effect on them personally? Has an increased focus on managing the economic self and wellbeing overtaken paying attention the psychological self and wellbeing? We see these questions as fertile areas for further research.

The stated limitations of this study present corollary research opportunities for the future. The sample size could be expanded to test if the identified sense of self characteristics from this study retained their presence and hierarchy in a larger group. The criteria employed in participant selection could be broadened to include CEOs of larger organizations and non-owner CEOs. Representative samples from different groups could be interviewed for example, individuals who founded social venture enterprises, to determine if the experience of leaders of non-profit organizations differs from their for-profit counterparts. Also, with a larger sample there may be an opportunity to differentiate subgroups of participants by sense of self characteristics.

The time period investigated could be condensed by focusing the interview questions on one of the four stages identified to draw the participants into more reflection on a specific phenomenon such as separation or transition. Alternatively, a longitudinal study could be conducted in which CEOs complete a self-rating of their sense of self with regard to a separation event and analyze what patterns might emerge. In addition, it would be interesting to interview the participants' spouses and work associates about the latter perspectives of the separation and transition process of the CEO.

Those individuals who retired vs. those that moved into new business endeavors could provide a more comparative model of sense of self on each group's separation and transition experience. Another differentiation would be those CEOs who sold their company to non-affiliated entities compared to those who sold to a successor generation or other family members.

Alternative methods of exploring the concept of sense of self could be employed through quantitative surveys. Constructing quantitative scales of valence might improve measurement of this concept. Also, it might be possible to develop correlations between specific coding groups by comparing gender, age, or family-owned businesses to non-family owned.

Finally, as we push a little further to speculate on the wider implications of this project for research on identity transitions and identity loss more generally, it occurs to us these CEOs are powerful people with high incomes who chose to step away from their work roles. Yet they were not well prepared for the transition and its impact on their identity. The result that they ended up with positive frames of mind at the end of the process may be a testament to their personal qualities and their resources. On the other hand, this should really be a cautionary tale for the rest of us, particularly those who have not chosen a career transition and do not have the financial and personal means to weather the change compared to the means available to these CEOs. We see this particular challenge a key area for future investigation in organizational psychology.

## Implications for practice

The participants in this study were each asked, “If you were going to speak to a group of private company owners about them leaving their company, what is the one thing you would want them to know from your experience?” From the voices of experience, 12 of the 16 interviewees referenced “the future” or “looking forward.” They described the importance of preparing for the future rather than waiting for it to unfold.

Resistance by the business owner, during preparation for a sale event, to attend to anything other than “the deal,” or making retirement plans that emphasize everyone else's needs are screens of personal uncertainty. Deflections of “Don't worry about me. I always land on my feet” or “I'll have so much time and money I can do whatever I want” are the first steps of a rappel without anchors.

Career consultants, human resource professionals, and others involved in a CEO's departure need to draw the executive's attention to the impending experience of shedding role sets and their accompanying identities. This might be accomplished by introducing the soon-to-be former CEO to a “guide” or a forum of guides. The model for this confidentiality-based forum exists in organizations such as Young Presidents Organization, World Presidents Organization, and Vistage. The difference is the forum described herein would be composed of former executives who can inform the CEO in familiar language about the losses and liberations awaiting them. The peer-to-peer component serves to offset “The one problem with self-assessment (which) is that people think they are quantitatively different from others, that whatever rules apply to other people do not apply to one's self” (Dunning, 2005, p. 166). The exiting CEO is not as unique as he or she may have been led to believe. For the transitioning CEO this means that an understanding and wariness of the closure of roles and associated role identities might be a helpful step in beginning the role exit process.

Recognizing the multiplicity of variables incumbent in a CEO's departure from his or her company, the application of the results of this study can serve as a foundation for planning. For example, questions utilized in this study might be modified and presented to a CEO during a Pre-Separation stage:

1. Can you describe for me your final week in the company? What do you imagine yourself doing each day?
2. What do you anticipate you will be doing 90 days after you exit?
3. Is there a metaphor that comes to mind that might describe your exit and subsequent 6 months?
4. What emotional highs or lows do you anticipate? Why?
5. Who do you anticipate will play important roles in your separation process? Why? Do they realize their importance and what you expect of them?
6. What does a successful transition look like for you? What does a failed transition look like?

These questions can raise the awareness of the CEO to impending issues and encourage forethought, planning, and preparatory steps for separation. For those individuals planning to shift to new roles with an acquiring company, the study results may serve to dispel unrealistic expectations and heighten awareness of potential problem areas. This same awareness might serve the purposes of an acquirer in structuring an employment agreement with the CEO that allows either party to accelerate the dissolution of an untenable work arrangement.

There did not appear to be an ideal profile of a CEO who was completely prepared for the experience of separating from his/her company. However, based on the participants' comments, there was substantial support that for the transitioning CEO, an awareness of the closure of roles and associated role identities might be a helpful step in beginning the role exit process.

## Conclusion

As a generation of baby boomers prepares to retire, among them are the top leaders for many of the organizations that drive the economies of the world. Our work here suggests several imperatives for both research and practice to more effectively address the separation from their organizations that awaits them.

## Author note

This paper is adapted from the unpublished doctoral dissertation of the first author.

### Conflict of interest statement

The authors declare that the research was conducted in the absence of any commercial or financial relationships that could be construed as a potential conflict of interest.
