# Supplementary material for: Voluntary transition of the CEO: owner CEOs' sense of self before, during and after transition
Source: Front Psychol. 2015 Oct 27;6:1633. doi: 10.3389/fpsyg.2015.01633 (PMC4621281; doi:10.3389/fpsyg.2015.01633)
Supplement: Supplementary file 1 [file DataSheet1.DOCX]

**Appendix A: Participant Profiles**

**Abe** was a multi-generation family CEO who bought the business from his father, who, in turn had bought the business from his father. As retirement approached, Abe took a direct and active role with his employees in establishing a leadership transfer process. “There wasn’t separation anxiety – separating myself from the company was just part of the plan...My identity as far as I was concerned, was what I was doing in the community.” With the completion of the exit transaction, Abe was “ready to move on …looking forward to being out of debt for the first time in my life.”

**Boris** founded his company at the age of 40. After 20+ years, “I just didn’t feel like going forward.” Proud of his accomplishments, “I enjoy what I perceive as respect for what I’ve done in business,” his identity and the company were connected but not intertwined. Boris is engaged in a transition into a new career that, “I think and hope will be interesting.” His perspective of the transition – “it has been seamless.”

**Carl** purchased the shares in his father’s company and proceeded to develop a highly successful enterprise founded on close relationships with his employees and a superior distribution system. Over time, the challenge of leadership diminished and so too did his interest in the business. The transition following the sale of his company has been rife with questions and unforeseen experiences – “If you’re going into retirement like I did – you’re going from being a big deal to just average…maybe it’s because I retired young, but I tend to have the feeling of being useless.”

**Donald** was opportunistic in identifying a business venture to purchase. Characterizing himself, “I’ve never been the classic Type-A firebrand,” he nevertheless led and grew the enterprise into an attractive purchase target by a major competitor. Prior to execution of a sale, Donald began a transition to more fully immerse himself in community leadership through his willingness to experiment with volunteer roles and a mindful awareness of his abilities.

**Ed** was the oldest entrepreneur in the study, founding his company in his 50s. The venture evolved into a highly successful family business. As retirement age approached -- “I was getting old…I made up my mind I was going to get out and give my kids an opportunity” -- he executed an ownership transfer plan that had been developed with the appropriate legal and financial advisors. Now retired, his perspective of his business career was captured by, “I don’t have any regrets. I have things to keep me occupied; I didn’t go brain dead (because I left my business).”

**Frank** built a small distribution business with less than $1M in revenue to a formidable competitor generating greater than $25M in sales. Faced with the classic business conundrum of growing to the next level (with all the inherent risks) or potentially being overtaken by the competition, Frank made his decision to sell. The commitment to exit his company “was very painful” literally, a physical decision: “it was a visceral feeling. I just felt I had to do it.”

**Greg**, founder of a small manufacturing firm that grew to $11M in revenues knew on “Day One” that his business was about an exit proposition. “I had never planned on this being a multigenerational business. It was never intended to pass on to sons or daughters.” Following a renegotiation of his employment contract and an early departure from the acquiring company, Greg embarked on a multiplicity of personal and professional roles: “I do a lot of different things. I spend time with my family. I garden when it’s warm. I run. I coach and mentor other people. I sort of have a smorgasbord.”

**Hank** held his ownership role for the shortest time period -- 6 years before selling his company. He was also the youngest former CEO in the study. He depicted his identity as the most closely aligned with his company: “I was invested in it physically, emotionally, spiritually.”

He described his present situation -- “There is a sense of floating, a sense of now what? What do I do to have meaning?”

**Ike**’s leadership philosophy was grounded in his military experience. Characterizing his management style as, “I play the team game, but I also own all the stock,” Ike built a nationally recognized service firm. He described his present outlook (sense of self) as a retired executive: “I keep what you might call a youthful attitude about the world of business and the nonprofit ways to help people.”

**Jim** purchased his business from his father. His anticipated exit strategy was a sale to the next generation in his family who had begun their apprenticeships. Approached with a selling opportunity that could not be ignored Jim felt he had to consider the offer’s implications beyond his personal desires. Following the execution of a successful sale, Jim stated, “I’m anxious to get on with my next phase. And I haven’t decided whether I’m going to let it be retirement or…pursue an opportunity.”

**Kate’s** entrepreneurial itch to be her own boss involved a career shift that culminated in an independent franchise operation with $10M of revenues. Following the sale of her enterprise, her transition to retirement was “a sense of freedom…we can do what we want.”

**Lucy** discovered rather than planned her way to business ownership. Initially unable to find employment as a social worker she took part-time assignments running high tolerance machinery in her father’s small, precision manufacturing company. Over time she developed her latent engineering aptitude. Eventually attaining the level of company CEO, she and her father were faced with the inevitable decision to sell: “the idea of selling, it was just a grief-wrenching thing, I just felt like somebody had died.” Today, following sale of the company, Lucy is engaged in new personal and professional roles. Recalling her experience as a business leader she stated, “When I think about some employees then I feel sort of a warm yearning in my upper chest for remembering the fact we were friends.”

**Mike’s** foray into business ownership was best summed by his own words: “most people, from the time I started, thought I was crazy.” After 25 years of competing with national companies in his sector he determined that it was time to sell. There was no looking back and second guessing. “I didn’t sit there and ponder, ‘Did I make a mistake?’ or get depressed about it or anything like that…If you’re going to constantly find fault with what you did, you’re going to go crazy.”

**Nick** described his beginnings as, “My parents gave me an education which was very nice of them, but I left the house with $200 in my pocket and a suitcase.” A financially successful entrepreneur and self-proclaimed man of action, “Guys like me, pretty much Type A personalities, willing to take risks,” Nick encountered a significantly less demanding post-CEO role. Nick immediately constructed a new identity in a smaller enterprise to counter the sense of loss. Acknowledging that “I would have been better off, personally, on reflection, just taking 6 months off, but the problem is, I've got some ongoing business responsibilities, too,” Nick continues his transition.

**Otto** gravitated to small business ownership from the C suite of a $3B business unit. Despite his extensive due diligence, he encountered a shrinking market, unfavorable currency valuations, and competition from better financed competitors. Forced to sell the business, when asked what thoughts or feelings he remembered from the close of the transaction he responded, “I had several emotions…the biggest one was a sense of failure…an admission of failure that I couldn’t find the right strategy to take the business forward and keep it.”

**Pat’s** story of exit from her company involved neither sale nor retirement but a planned close of her enterprise. Recognizing in her late 20s that, “I always wanted to have my own business…It was an instinctive feeling…I knew that I could take the risk,” she established a niche service firm that enjoyed a national reputation. As she approached the decision to close the company that was intentionally built around her -- “I was the business. The product was me” -- she methodically informed staff and customers of her plans. “A year before I was going to close the business down, I told my staff…I really knew what I wanted if I listen(ed) to myself.”

## Appendix B: Interview Guide

This is an interview about your experience of separation and transition from your company. We are going to ask you questions about three different time periods: the period during your separation; the transition period following the separation; and today. We are interested in learning about both what you thought and what you felt, in other words your sense of self, during those periods. Everything you say is confidential and you are welcome to end the interview at any point if you wish. Do you have any questions before we begin?

1. Please begin by telling me some background about your role as CEO. How did you come to the position of ownership?

1. Now, can you tell me about the circumstances in which you exited your role of CEO? Why did you leave?

1. What it was like for you to leave your position as CEO?

Probe: How did you think of yourself during and following the separation? Probe: Is there any metaphor that comes to mind that might describe your exit and transition?

1. Can you describe for me when you knew the exit and separation were complete or coming to a closure?

Probe: What thoughts and feelings did you experience?

1. What were some of the emotional highs or lows that you felt during the exit? Probe: What about during the transition following the separation?

1. Do you recall any particular influences on you during your exit or transition? Probe: How would you describe them?

1. During the exit or transition, what would you say was your greatest personal challenge?

Probe: How did you deal with that challenge?

1. As you look back at your exit and transition experience, how would you describe your self-perception or identity during both the exit and the transition?

Probe: Has that self-perception or identity changed to the present and if so, how?

1. What would you say that you learned about yourself from the exit and transition experience?

1. Following your exit did you actively seek any new roles? If so, what were they?

Probe: How did you select them?

1. When someone asks you now “What do you do?” What is your response? Probe: Describe the feelings associated with the response.

Probe: Is the response or feelings different now that at the time of exit? How?

1. What would you like to add to this interview that is important to you and we have not addressed?
